# Apical sling for laparoscopic sacrohisteropexy in a young virgin patient with joint hypermobility syndrome

**DOI:** 10.1590/S1677-5538.IBJU.2018.0746

**Published:** 2020-01-13

**Authors:** Alcidézio Farias Santana, Raquel Doria Ramos Richetti, Susane Mey Hwang, Tatenda Nzenza, Luis Gustavo M. Toledo

**Affiliations:** 1 Departamento de Cirurgia, Hospital Irmandade da Santa Casa de Misericórdia de São Paulo, SP, Brasil; 2 Departamento de Ginecologia, Hospital Maternidade Vila Nova Cachoeirinha, São Paulo, SP, Brasil; 3 Departamento de Uroginecologia, Hospital Maternidade Vila Nova Cachoeirinha, São Paulo, SP, Brasil; 4 Austin Health, Urology Heidelberg, Victoria, Australia; 5 Departamento de Urologia, Faculdade de Medicina da Santa Casa de São Paulo, SP, Brasil

## Abstract

**Introduction:**

We are faced with a young patient with uterine prolapse and urinary difficulties due to Joint Hypermobility Syndrome, a congenital collagen disease that predisposes woman to the development of pelvic organ prolapse. The patient had urinary difficulty requiring standing and bowing to reduce prolapse and then start urination. This video demonstrates that videolaparoscopic technique is feasible for the treatment of uterine prolapse in young and sexually virgin woman.

**Materials and Methods:**

We separated the bladder from vagina and opened the peritoneum anterior to the uterus. Next, we attached the sigmoid colon to the left abdominal wall in order to better expose the promontory. We then opened the peritoneum posterior to the uterus and medially tunnelled the right uterosacral ligament, transfixing the broad ligament and passing the end of a polypropylene mesh through this tunnel to the posterior region of the uterus. The same maneuver was performed on the other side so that the mesh surrounded the anterior portion of the cervix while its two extremities were posterior to the uterus. The mesh was fixed on the anterior surface of the uterine cervix and its two extremities were fixed to the promontory in the anterior longitudinal ligament of the spine. Finally, we closed the peritoneum.

**Results:**

Uterine prolapse was corrected, with good recovery.

**Conclusions:**

Videolaparoscopic technique is feasible for correction of uterine prolapse, being effective and safe in virgin woman.

## ARTICLE INFO

**Available at: http://www.intbrazjurol.com.br/video-section/20180746_Santana_et_al**

**Int Braz J Urol. 2020; 46 (Video #2): 139-139**

